# Complement in Hemolysis- and Thrombosis- Related Diseases

**DOI:** 10.3389/fimmu.2020.01212

**Published:** 2020-07-10

**Authors:** Shanshan Luo, Desheng Hu, Moran Wang, Peter F. Zipfel, Yu Hu

**Affiliations:** ^1^Institute of Hematology, Union Hospital, Tongji Medical College, Huazhong University of Science and Technology, Wuhan, China; ^2^Department of Integrated Traditional Chinese and Western Medicine, Union Hospital, Tongji Medical College, Huazhong University of Science and Technology, Wuhan, China; ^3^Department of Infection Biology, Leibniz Institute for Natural Product Research and Infection Biology, Hans Knöll Institute, Jena, Germany; ^4^Friedrich Schiller University, Faculty of Biological Sciences, Jena, Germany

**Keywords:** complement, hematological disorder, anti-complement agent, platelet, hematology

## Abstract

The complement system, originally classified as part of innate immunity, is a tightly self-regulated system consisting of liquid phase, cell surface, and intracellular proteins. In the blood circulation, the complement system, platelets, coagulation system, and fibrinolysis system form a close and complex network. They activate and regulate each other and jointly mediate immune monitoring and tissue homeostasis. The dysregulation of each cascade system results in clinical manifestations and the progression of different diseases, such as sepsis, atypical hemolytic uremic syndrome, C3 glomerulonephritis, systemic lupus erythematosus, or ischemia–reperfusion injury. In this review, we summarize the crosstalk between the complement system, platelets, and coagulation, provide integrative insights into how complement dysfunction leads to hemopathic progression, and further discuss the therapeutic relevance of complement in hemolytic and thrombotic diseases.

## Introduction

The complement system is an ancient and critical effector mechanism of the innate immune system, which consists of central components of the whole cascade (C1 to C9), regulators and inhibitors, proteases and newly assembled enzymes, multiple activation products, and receptors for complement components and their products (1). Based on the different mechanisms for its initiation, the complement system is classified into three pathways (the alternative, lectin, and classical pathways, short as CP, LP, AP) that merge at the level of C3 cleavage. Being a central immune surveillance system, complement can be activated within seconds upon infection or stimulation. Complement activation leads to the generation of anaphylactic peptides, cytolytic compounds, and antimicrobial compounds. These generated molecules in turn activate pro-inflammatory mediators and recruit effector cells, thereby providing an immediately acting barrier against invading microbes or modified self-cells, including tumor cells, in a self-controlling manner (2, 3). In addition, complement also functions in regulation of adaptive immunity, mediation of cell integrity, and tissue homeostasis (4).

Apart from the complement cascade, the coagulation and the fibrinolytic systems are also enzymatic cascades existing in the blood. The coagulation cascade and fibrinolytic system plus platelets compromise the hemostasis system (5). Hemostasis is classically defined as the cessation of bleeding in the body and takes place in a highly organized and time-dependent manner at the site of vascular injury. The coagulation cascade comprises the intrinsic pathway and the extrinsic pathway. Activation of coagulation cascade *via* each pathway leads to fibrin formation. Platelets, also called “thrombocytes,” have no nucleus and are fragments of the cytoplasm derived from the megakaryocytes of the bone marrow that enter the blood circulation (6). Platelets have a major function in repairing vascular damage and stopping acute bleeding. When the endothelium is broken, platelets are immediately activated by different factors, such as collagen and tissue factors (7). The activated platelets and fibrin are then cross-linked together *via* GpIIbIIIa, forming thrombus. Meanwhile, platelets also contribute to thrombus formation in atherosclerosis, venous thrombosis, myocardial infarction, disseminated intravascular coagulation (DIC), and many other pathological conditions.

An expanding body of evidence suggests multiple interactions between the hemostatic system and innate immunity exist, especially the complement system. Both systems consist of fluid phase factors distributed in the blood, in which these factors form a close network, interact with each other, tightly self-regulate, and mediate immune surveillance and tissue homeostasis (8). However, the dysregulation of any component in both systems results in pathological conditions and clinical manifestations of the diseases with critical thrombotic or inflammatory complications, such as sepsis, systemic lupus erythematosus, or ischemia-reperfusion injury (9).

In this review, we first give an overview of the close interaction between complement and hemostatic networks, then provide a deep insight into the roles of complement in hematologic disorders and further discuss current complement-based immunotherapy in treating such disorders. This overview is crucial in understanding hemopathic pathology and guiding the development of complement-based diagnostic tools and valuable therapies to improve the clinical management of patients with hemopathic conditions.

## The Crosstalk Between Complement and Hemostasis

The complement system is genetically derived from the serine protease reaction cascade encoded by the same ancestor gene as coagulation factors. In blood circulation, complement forms close networks with not only platelets but also the coagulation and fibrinolytic systems, participating in a wide range of biological functions. The common roles of these systems are to present the first defense line against infectious microbes that enter the bloodstream and blood circulation, to initiate repairment after tissue damage, and to cause adverse reactions either maintaining homostasis or resulting in severe disorders (10). In this part, we summarize the interaction network between complement, platelets, and coagulation cascade.

### The Interplay Between Complement and Platelets

Hamad et al. reported that the complement system activated platelets in various ways, while thrombin-activated platelets in turn activated complement cascade, which forms a potential self-strengthening cycle (10), indicating a close interplay between the complement system and platelets.

#### The Effect of the Complement System on Platelet Activation

**Figure 1:**
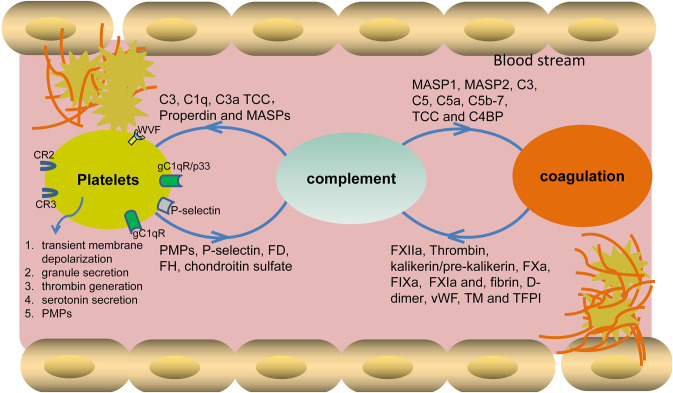
Cross-interaction of complement with platelets and the coagulation system. Different complement components bind to platelet surface receptors and also closely interplay with coagulation factors, leading to the activation of platelets and also the initiation of coagulation cascade. In turn, the activated platelets and coagulation factors also assist complement activation by binding or interfering with different components. TCC, terminal complement complex; MASPs, mannose-binding lectin-associated serine proteases; FD, factor D; FH, factor H; PMPs, platelet microparticles, VWF, von Willebrand factor.

An early *in vitro* report showed that thrombin-mediated platelet aggregation and serotonin secretion are highly enhanced by the combination of C3 and terminal complement complex (TCC). In this process, Polley et al. found that thrombin associated with the platelet membrane presumably initiated C3 convertase formation in a way different from the known classic or alternative mechanisms. The formed C3 convertase entered the known complement sequence at the C3 stage and proceeded to activate the terminal components through C5 to C9, which may enhance the uptake of the C3 and TCC complex by platelets. In turn, the activated complement system on the platelet surface, as a combination of C3 and TCC, highly enhanced platelet aggregation and serotonin secretion (11, 12). Another report showed that TCC induced membrane microparticle formation, thereby exposing the binding sites for factor Va and serving as a basis for the proteolytic generation of thrombin (13). Furthermore, both *in vivo* and *in vitro* data have shown that TCC-mediated stimulation of platelets causes transient membrane depolarization (14), granule secretion (15), induction of phosphatidylserine, and platelet-catalyzed thrombin generation, affecting platelet activation and coagulation initiation (13, 16, 17) (Figure 1). In addition, Koelm et al. reported that surface-bound C1q, by interacting with the von Willebrand factor (VWF), induced platelet rolling (18). C1q was also shown to bind gC1qR/p33 or gC1qR on platelet surfaces, thereby initiating platelet activation, a process that can further induce the aggregation of platelets *via* a P-selectin-dependent pathway (19–21). *In vivo* data showed that C3, independently of TCC formation, played specific roles in platelet activation. *C3*^−/−^ mice have prolonged bleeding time and diminished platelet activation, further proving a direct link between complement and platelet activation (22, 23). Besides, the anaphylatoxin C3a and its derivative C3adesArg directly induced platelet activation and aggregation (24). In comparison to *C3*^−/−^ mice, *C5*^−/−^ mice have no apparent defect in platelet activation, platelet deposition in the vessel wall, and the initial hemostasis (22).

#### The Effect of Platelets on Complement Activation

Apart from the above-mentioned effects of complement on platelet activation, platelets also initiate/regulate complement activation *via* different secreted or surface-expressed factors. As for initiating complement activation, platelet-expressed P- selectin activates complement either on its own or by fixing C3b from spontaneous basal plasmatic C3 cleavage (25–27). Further platelets, by secreting chondroitin sulfate, bind C1q or factor D, thereby initiating local complement activation (28–30). As for regulating complement activation, platelets, by secreting Factor H from alpha-granules, either regulate C3 convertase activity or modulate C1q effects *via* CR3 (31, 32). However, contradictory data showed that Factor H was present throughout the cytoplasm and on the surface of normal resting platelets but with no evident concentration of Factor H in alpha-granules, lysosomes, or dense granules (33). In addition, platelet-expressed VWF was recently identified as a novel complement regulator that can protect endothelial cells from injury by down-regulating complement activation (34). Furthermore, a platelet, by secreting chondroitin sulfate, binds complement regulators C1 inhibitor (C1INH), C4b-binding protein (C4BP), and Factor H, thereby blocking complement activation on its surface (10, 35).

Aside from the above-mentioned close interplay between complement and platelets, a growing body of evidence indicates that complement activation influences platelet-associated pathologies, such as idiopathic thrombocytopenic purpura (ITP) (36), atypical hemolytic uremic syndrome (aHUS) (37, 38), and paroxysmal nocturnal hemoglobinuria (PNH) (39, 40). Currently, such diseases are treated with anti-complement therapeutics, such as Eculizumab, H17/3E7, and TNT003, controlling the pathophysiological processes that are driven by complement over-activation (details will be discussed further in section Therapeutic Relevance of the Complement System).

### The Interplay Between Complement and Coagulation

The complement and coagulation cascades are evolutionarily related enzymatic cascades in blood circulation. They are linked in their activation mechanisms and influence innate immune functions following tissue injury. Early reports showed that the levels of complement activation products in normal human serum are much higher than in anti-coagulated blood, indicating the progression of complement activation upon blood clotting (41).

#### The Effect of the Complement System on Coagulation Cascade

The complement system activates coagulation cascade via multiple factors (Figure 1). *In vitro* data showed that activated MASP-1 cleaved fibrinogen, fXIII, and TAFI, further promoting fibrin cross-linking (42, 43), while MASP-2 participated in the activation of thrombin and the subsequent generation of fibrin (44). Further, the complement activation products C5a and TCC trigger tissue factor expression and activation in both human endothelial cells and neutrophils, which results in the activation of the extrinsic coagulation pathway (45, 46). C5a was also shown to induce the up-regulation of PAI-1, thereby promoting a local procoagulant effect on mast cells (47, 48). Additionally, TCC can cause an increase of blood thrombogenicity by simultaneously inducing procoagulant and antifibrinolytic proteins and inhibiting natural anticoagulants (49, 50). Besides the above-mentioned roles of the complement activation products, the complement regulators also display obvious effects on coagulation. For example, C4b binding protein (C4BP), by binding to protein S, affects natural anticoagulation *in vitro* (51). Another complement regulator, C1 inhibitor, also shows an inhibitory effect on the activity of fXIIa, fXIa, and kallikrein in the coagulation system (52–54). Importantly, Subramaniam et al., by using an *in vivo* mouse model, showed that C5^−/−^ and C3^−/−^ mice had longer tail bleeding times and were less susceptible to thrombosis, further proving that complement plays important roles in the progression of coagulation (22).

#### The Effect of Coagulation Cascade on Complement Activation

The interplay between complement and coagulation cascades occurs in both directions. The coagulation factors can also activate complement cascade at different levels. For the initiation of complement activation, Factor XII binds to the complement C1 or the globular C1q receptor (gC1qR), initiating CP activation (55, 56). Fibrin and the plasmin-generated fibrin fragment (D-dimer) in plasma bind and activate MASP-1 and MASP-2, leading to LP activation. Furthermore, pre-kallikrein cleaves Factor B and activates C1s, thereby activating both AP and CP (57). In terms of C3 activation, the interaction of fibrinogen/fibrin with the MASPs also modulates the activation of C3 and C4, as well as the surface deposition of C3b and C4b (58). Meanwhile, kallikrein can cleave not only complement factor B but also C3 and C5 (53, 59, 60). Also, on the C5 level, *in vivo* data showed that thrombin cleaved C5 in the absence of C3 (61). Further investigation revealed that, besides thrombin, several other factors (i.e., FIXa, FXa, FXIa, and plasmin) can also cleave C3 and C5, leading to C3a and C5a generation, respectively. What is more, thrombomodulin and tissue-factor pathway inhibitor participate in complement regulation (62–65).

Besides the above-mentioned two-way interplay between the coagulation and complement cascades, NETosis appears to be a third important player involved in the complement-coagulation interaction, forming a triangular relationship to protect the host against pathogens. Activated complement proteins stimulate NET formation, and NETs, in turn, serve as a platform for complement activation. Furthermore, NETs act as a scaffold for thrombus formation during coagulation (66). Taken together, all of the close interplay between coagulation and complement explains why the association of both systems with several clinical inflammatory and thrombotic conditions exists.

## Complement in Hemolytic and Thrombotic Diseases

**Figure 2:**
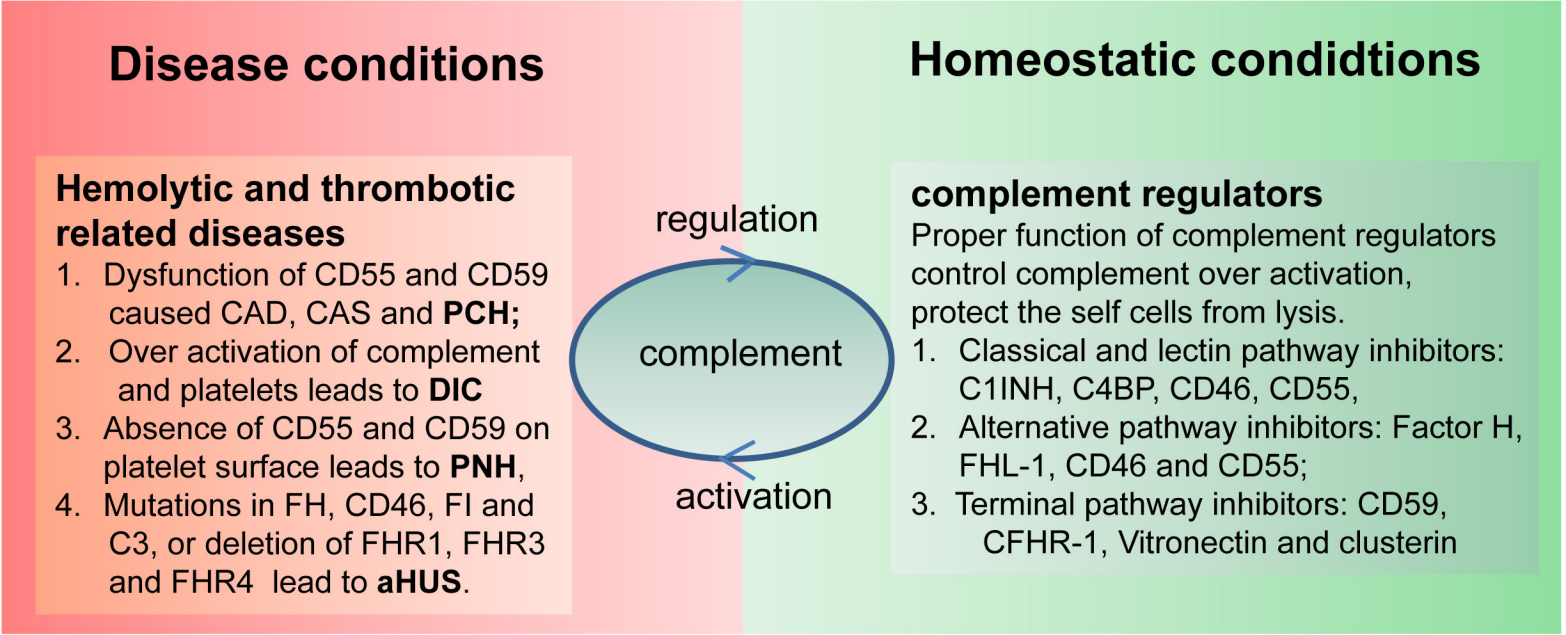
Link between complement and hematological disorders. Complement, on the one hand, can protect the host from infection and maintain body homeostatic conditions, and, on the other hand, it may also cause hematological disorders due to uncontrolled activation or utilization of complement regulators. C1INH, C1 inhibitor; FHL-1, factor H-like protein 1; C4BP, C4 binding protein; AP, alternative pathway; CP, classical pathway; LP, lectin pathway; TP, terminal pathway; CFHR1, complement factor H related protein 1; TCC, terminal complement complex; MASPs, mannose-binding lectin-associated serine proteases; FB, factor B; FD, factor D; FP, factor P.

Complement activation plays an essential role in controlling infection and maintaining homeostasis, whereas complement dysfunction is associated with the pathogenesis of multiple hemolytic and thrombotic diseases (Figure 2). Cross-interplay of the complement and hemostatic systems could be a key mediator of “thrombo-inflammation” (67). Abundant evidence demonstrated that complement hyperactivation is correlated with thrombosis and the development of multiple organ failures. This section aims to provide an integrative overview of the mechanisms underlying the interactions between complement and hemostasis in disease pathology.

### Complement in Autoimmune Hemolytic Anemia

Autoimmune hemolytic anemia (AIHA) is a collective term for several diseases. It is characterized by autoantibody-mediated destruction of red blood cells. Complement activation in AIHA may exacerbate extravascular hemolysis and may also occasionally result in intravascular hemolysis. In particular, the subgroup primary cold agglutinin disease (CAD), secondary cold agglutinin syndrome (CAS), and paroxysmal cold hemoglobinuria (PCH) are entirely complement-dependent disorders, whereas warm-antibody AIHA is only partially related to complement activation (68).

In CAD, autoimmune disorders are often associated with CP hyperactivation (69). The major subtype of cold agglutinin (CA) is the IgM subtype, which accounts for 90% of the total cases (29). IgM binds to erythrocytes at the end of circulating limbs and then activates CP, resulting in C3 cleavage (30). When returned to the central part of the circulation with a body temperature of 37°C, IgM becomes dissociated from the cell surface, but C3b remains bound to red blood cells. These C3b opsonized red blood cells bind to complement receptors on systemic macrophages. Afterwards, they are cleared in the liver to mediate extravascular hemolysis. In homeostatic conditions, the expression of complement regulators CD55 and CD59 on erythrocyte membranes blocks complement activation and protects the erythrocytes from lysis. However, in severe conditions with infection, surgery and autoimmune diseases, intravascular hemolysis caused by the over-activation of TP may occur due to the dysfunction of these two surface-expressed regulators (28, 70). Current therapy for IgM-mediated autoimmune hemolytic anemia mainly aims to decrease auto-antibody production. However, most of these treatments require time to become effective and also will neither stop immediately ongoing complement-mediated hemolysis nor prevent hemolysis of transfused red blood cells. Therefore, direct inhibition of the complement system might be a suitable approach to halt or at least attenuate ongoing hemolysis, which helps to improve the recovery of red blood cell transfusion in autoimmune hemolytic anemia. In recent years, several complement inhibitors have become available in the clinic with proven efficacy in autoimmune hemolytic anemia, which will be discussed in section Therapeutic Relevance of the Complement System.

CAS is different from CAD in terms of etiology and the locality of B cells, but it is characterized by the same complement-dependent hemolysis mechanism (71). Furthermore, the hemolysis of PCH is entirely complement-dependent. Polyclonal IgG antibodies bind to the P antigen on the erythrocyte surface but do not agglutinate erythrocytes. This P-antigen-antibody complex is a very strong complement activator, which leads to complete over-activation of CP and TP, mediates massive intravascular and extravascular hemolysis, and causes sudden onset of the disease.

In contrast, auto-antibodies to warm-AIHA (w-AIHA) mostly belong to the IgG class (72). In up to 50% of w-AIHA, a direct antiglobulin test is positive for complement fragments, and the most common one is C3d (73). Another autoimmune disease characterized by platelet destruction and thrombocytopenia is immune thrombocytopenia purpura (ITP) (74). Thrombocyte destruction in ITP is mediated by multiple immune mechanisms. Interestingly, some ITP patients have increased complement activation, indicating that complement also takes part in ITP progression. However, how the complement system interferes with ITP progression remains unclear.

### Complement in Disseminated Intravascular Coagulation

Disseminated intravascular coagulation (DIC) is characterized by an increased incidence of thrombin formation or hemorrhagic diathesis due to the consumption of platelets and coagulation proteins in the circulation. The most common cause of DIC is sepsis, being characterized by an over activation of complement and platelet activation. Others reported that mannose-binding lectin (MBL) and/or MASP-1/3 were involved with hemostasis following injury. *Staphylococcus aureus*-infected MBL null mice developed DIC with the characteristic of elevated IL-6 levels in blood. Infected MBL null mice also developed liver injury, suggesting that MBL deficiency may develop into DIC and organ failure during infectious diseases (75). Furthermore, Zhao et al. reported, on the basis of 276 patient samples, that the complement components were modified in sepsis patients with DIC. The statistical data showed that C3, TCC, and MBL were significantly increased in septic patients with DIC (76).

### Complement in Paroxysmal Nocturnal Hemoglobinuria

Paroxysmal nocturnal hemoglobinuria (PNH) is an acquired syndrome characterized by intravascular hemolysis, thrombosis, and bone marrow failure (77). Thrombosis is the most urgent indication for therapies and the leading cause of PNH death. The reasons for the thrombosis tendency in PNH are multifactorial. The absence of two glycosylphosphatidylinositol-anchored complement surface proteins, CD55 and CD59, on the platelet's surface of PNH patients leads to the formation of pre-thrombotic particles. CD55 regulates the formation of C3 and C5 convertases by binding to C3b and C4b and preventing the amplification of complement cascade, while CD59 prevents C9 polymerization and TCC formation. The absence of these two regulators leads to uncontrolled complement activation and lysis of the cell membrane, which accounts for hemolysis and other PNH manifestations (78).

Meanwhile, C5a was clearly shown to induce inflammation and thrombosis by inducing the release of inflammatory cytokines, such as IL-6, IL-8, and TNFα (46). Furthermore, Fibach et al. show that oxidative stress, in conjunction with activated complement, may cause the underlying symptoms of PNHs, such as intra- and extravascular hemolysis and thrombotic complications. Besides dysfunction of the complement system, other deficiencies such as in heparin sulfate and GPI-anchored common receptor are also presumed to favor thrombosis in PNH (79, 80). However, it is not clear which mechanism has the greatest impact on PNH thrombosis. Currently, the most effective strategy to prevent PNH thrombosis is complement inhibition on TP (81). Eculizumab is indicated for the treatment of acute thromboembolic events.

### Complement in Atypical Hemolytic Uremic Syndrome

Atypical hemolytic uremic syndrome (aHUS) is a type of thrombotic microangiopathy characterized by glomerular endothelial damage, thrombosis, and mechanical hemolysis. Both genetic and acquired abnormalities of the complement system can cause aHUS progression (82). Complement activation is normally controlled by regulatory proteins, such as Factor H and FHL-1 in plasma and CD46 on the cell surface. Approximately 70% of patients with aHUS have mutations in genes that regulate the AP activation because the pathogenesis of aHUS is strongly correlated to the AP dysfunction (82–85). Most patients have one defective allele and one intact allele of Factor H (86), which occurs in the carboxy-terminal surface recognition region and leaves the complement-regulatory region of the amino-terminus intact. These heterozygous mutations of Factor H gene reduce its surface binding to erythrocytes, weakening the protective surface property, and thereby leading to uncontrolled complement activation. The amplified local complement activation causes erythrocyte lysis and host tissue damage. Besides the mutation of fluid-phase regulator Factor H, heterozygous mutations of CD46 also predispose to aHUS (87). Further, mutations in factor I and C3 were identified in aHUS. In addition, Abarrategui-Garrido et al. reported that a novel genetic mutation in CFHR1 is highly associated with aHUS (88). Importantly, in aHUS patients, deletions of CFHR1, CFHR3, and CFHR4 lead to the development of Factor H autoantibodies (89). These autoantibodies bind to and block the C-terminal recognition region of Factor H, leading to uncontrolled AP complement activation.

## Therapeutic Relevance of the Complement System

In the last decade, the complement system has been increasingly focused on since its inappropriate or uncontrolled activation is closely related with many diseases, such as thrombotic and hemolytic diseases and autoimmune diseases. The application of anti-complement agents in the clinic represents a major technical achievement, suggesting a novel etiologic treatment for different human diseases (90).

Eculizumab, the first anti-complement agent, is a monoclonal antibody that binds to C5, interferes with C5 cleavage, and thereby inhibits TP activation (91). Eculizumab is approved for PNH, aHUS, and Myastenia gravis, with potential impact on the severe clinical course. This TP inhibitor was later also proved efficacious for the treatment of severe idiopathic warm AIHA. With more and more interest in the complement therapies, different subtypes of C5 inhibitors with higher specificity were generated, such as Ravulizumab (A.LXN1210) and ALN-CC5 (Table 1). Ravulizumab, being a second-generation of C5-specific monoclonal antibody, provides immediate, complete, and sustained C5 inhibition. Ravulizumab binds to C5 with a higher affinity than Eculizumab, inhibiting the formation of C5a and C5b, thereby blocking the occurrence of complement activation and hemolysis, and achieving better readout when treating patients with PNH and aHUS (98). ALN-CC5 is another TP inhibitor that targets C5 via RNA interference. ALN-CC5 can significantly reduce the C5 level and inhibit complement activity. Importantly, Hill et al. reported that patients with PNH have good tolerance to ALN-CC5 and prolonged duration of drug efficacy (99).

**Table 1:** The main complement-related immunotherapies used in hematological disorders.

**Complement therapies**	**Targeted factors**	**Diseases to be treated**	**Readout**	**Molecular nature**	**References**
TNT003	C1s	CAD, ITP	Higher specificity, narrow effect	Monoclonal antibody	(69, 92)
Sutimlimab	C1s	CAD	Higher specificity, narrow effect	Monoclonal antibody	(93)
Cp40	C3b, C3d	PNH	Higher specificity, narrow effect	Cyclic polypeptide	(94)
PEG - Cp40	C3b, C3d	PNH	Higher specificity, narrow effect	Cyclic polypeptide	(94)
TT30	iC3b/C3d	PNH, AIHA	Higher specificity, narrow effect	Fusion protein	(95)
CRIg-L-FH	C3b/C3bi	PNH	Efficiently protected aberrant erythrocytes	Fusion protein	(96)
CRIg-FH	C3b/C3bi	PNH	Efficiently protected aberrant erythrocytes	Fusion protein	(96)
H17/3E7	C3b/C3bi	PNH	Efficiently inhibits erythrocytes lysis	Monoclonal antibody	(97)
Eculizumab	C5	PNH, aHUS, wAIHA	First generation of C5 inhibitor, broad effect	Monoclonal antibody	(91)
Ravulizumab	C5	PNH, aHUS	High affinity, better effect	Monoclonal antibody	(98)
ALN-CC5	C5	PNH	Good tolerance and long duration	siRNA	(99)

*CAD, cold agglutinin disease; ITP, immune thrombocytopenia purpura; PNH, Paroxysmal nocturnal hemoglobinuria; AIHA, Autoimmune hemolytic anemia; aHUS, Atypical hemolytic uremic syndrome. The order was classified according to the target factors of the whole complement cascade*.

However, C5 inhibitors have broad effects on the TP of the complement system, lacking specificity. Widespread inhibition of the complement cascade may jeopardize patient health due to increased susceptibility to infections. Thus, the development of pathway-specific complement inhibitors has been a long-lasting goal over the past few decades. Compstatin, Compstatin 40 (Cp40), and its long-acting analog, polyethylene glycol(PEG-Cp40) are newly designed complement inhibitors that display inhibition on the upstream of the complement cascade (92). Compstatin is a cyclic fibrin polypeptide composed of 13 amino acids that binds to C3 and C3b. At present, the compstatin-related inhibitors used for the treatment of patients with PNH are mainly Cp40 and PEG-Cp40 (94).

Another type of pathway-specific inhibitor is recombinant fusion protein, which includes TT30 and CRIg-L-FH/CRIg-L-FH. TT30 is a 65-kDa recombinant human fusion protein, consisting of the iC3b/C3d-binding region of complement receptor 2 and the inhibitory domain of the regulator Factor H, that can efficiently block AP activation. Risitano et al. confirmed that TT30 completely inhibited erythrolysis in PNH patients and C3b-mediated extravascular hemolysis in a dose-dependent manner (95). In addition, TT30 effectively inhibits AP-mediated C3b deposition on the erythrocyte membrane in a CR2-dependent manner, blocks the formation of TCC, and inhibits both intravascular and extravascular hemolysis of AIHA (95). CRIg-FH and CRIg-L-FH are novel CRIg-targeted complement inhibitors that are designed by connecting the functional domains of CRIg and Factor H. CRIg-L-FH is slightly more potent than CRIg-FH. Both regulators dramatically inhibited both AP- and CP-mediated hemolysis and successfully eliminated the deposition of C3b/iC3b, thereby efficiently protecting aberrant erythrocytes of PNH patients (96).

The third type of pathway-specific inhibitor is monoclonal antibodies, including TNT003, Sutimlimab, and H17/3E7. TNT003, as shown by Peerschke et al., is a mouse-derived monoclonal antibody that targets serine proteinase C1s, inhibits complement deposition and the formation of TCC complexes, and thus prevents intravascular and extravascular hemolysis in CAD patients (69). Statistical analysis showed that when TNT003 was applied in ITP patients, plasma C4d, C3b, and C5b-9 deposition were decreased dramatically (92), suggesting a possible therapeutic effect of TNT003 on ITP. Sutimlimab, another monoclonal antibody that binds C1s, quickly blocks hemolysis, corrects anemia, and eliminates the need for blood transfusion in patients with CAD. Based on these data, sutimlimab was given a breakthrough treatment designation by the U.S. Food and Drug Administration for treatment of this condition (93). Lindorfer et al. reported that the monoclonal antibody H17/3E7 was directed to combine with C3b/iC3b to effectively inhibit the lysis of erythrocytes in PNH patients. By combining with C3 and C3b, H17/3E7 can inhibit the formation of C3 and C5 convertase, thereby effectively inhibiting AP activation, whereas CP activation is not affected (97). These inhibitors have been more and more widely used for the treatment of thrombotic and hemolytic diseases, indicating that anti-complement agents are potential therapeutic drug candidates for a range of complement-mediated diseases.

## Concluding Remarks and Perspectives

A large amount of experimental and clinical evidence shows that complement closely interplays with hemostasis systems and participates in many important biological functions, while dysfunction of the complement system directly or indirectly interferes with many hemopathic progressions. Thereby, complement-related therapies have become more and more into focus. However, current existing anti-complement agents display a broad effect but lack specificities, which may increase patient susceptibility to infections due to general inhibition of the overall complement-mediated effector functions. New therapies for precisely targeting complement are on the way and are urgently needed. One of the great challenges in this aim is to clarify the disease pathogenesis and find out which complement component's dysfunction leads to hemopathic progression and how.

## Author Contributions

SL contributed by collecting data, designing the structure, writing, and review. DH contributed to collecting data and writing. MW contributed to collecting data. PZ contributed to writing, reviewing, and designing the topic. YH contributed to designing the topic, writing, and reviewing. All authors contributed to the article and approved the submitted version.

## Conflict of Interest

The authors declare that the research was conducted in the absence of any commercial or financial relationships that could be construed as a potential conflict of interest.

## Abbreviations

TCC: terminal complement complex
AP: alternative pathway
LP: lectin pathway
CP: classical pathway
C3: complement factor 3
ITP: idiopathic thrombocytopenic purpura
HUS: hemolytic uraemic syndrome
aHUS: atypical hemolytic uremic syndrome
PNH: paroxysmal nocturnal hemoglobinuria
MASP2: mannose-binding lectin-associated serine protease 2
AIHA: autoimmune hemolytic anemia
CAD: cold agglutinin disease
CAS: secondary cold agglutinin syndrome
PCH: paroxysmal cold hemoglobinuria
DIC: disseminated intravascular coagulation.
